# Comparative evaluation of propolis nanostructured lipid carriers and its crude extract for antioxidants, antimicrobial activity, and skin regeneration potential

**DOI:** 10.1186/s12906-022-03737-4

**Published:** 2022-10-03

**Authors:** Ola M. Elkhateeb, Mohamed E. I. Badawy, Ahmed E. Noreldin, Howaida M. Abou-Ahmed, Mahmoud H. El-Kammar, Hoda A. Elkhenany

**Affiliations:** 1 Department of Surgery, Faculty of Veterinary Medicine, Alexandria University, Alexandria, 22785 Egypt; 2 Department of Pesticide Chemistry and Technology, Faculty of Agriculture, Alexandria University, El-Shatby, Alexandria, 21545 Egypt; 3 Department of Histology and Cytology, Faculty of Veterinary Medicine, Damanhour University, the Scientific Campus, Damanhour, 22511 Egypt

**Keywords:** Herbal extract, Propolis, Nanoparticles, Wound healing, Antioxidant, Carbopol gel

## Abstract

**Background:**

Propolis extracted from beehives has been conferred with natural antimicrobial and antioxidant properties. Hence, it has been recommended as a wound healing therapy. This study investigated the additive value of nanotechnology to the herbal extract, (propolis rebuts), after which we examined its efficacy in wound healing.

**Methods:**

Propolis nanostructured lipid carriers (NLCs) were first prepared using the emulsion-evaporation-solidification method at three concentrations. Then, we compared their flavonoid and phenolic contents and phenolic contents. Their antioxidant, antibacterial, and antifungal effects were also investigated after which, the skin regenerative capacity of propolis-NLCs was assessed using full-thickness skin wounds in rabbits.

**Results:**

This study showed that propolis-NLCs had increased the phenolic and flavonoid contents compared to the raw propolis extract (EXTR) (9-fold and 2-fold, respectively). This increase was reflected in their antioxidant activities, which dramatically increased by 25-fold higher than the propolis-EXTR. Also, propolis-NLCs exhibited a 2-fold higher potent inhibitory effect than propolis-EXTR on Gram-positive bacteria (*Bacillus subtilis* and *Staphylococcus aureus*), Gram-negative bacterium (*Salmonella spp*.), and fungus (*Candida albicans*) microbes (*p* < 0.0001). Investigations also revealed that treatment of full-thickness skin injuries with propolis-NLCs resulted in significantly higher wound closure compared to propolis-EXTR and the control after two weeks (*p* < 0.0001).

**Conclusion:**

With a prominent broad-spectrum antibacterial effect propolis-NLCs exhibited higher skin regenerative potency than propolis-EXTR. We also highlighted the additive impact of nanotechnology on herbal extract, which accounted for the increased flavonoid content and hence a better antioxidant and antimicrobial effect and propose it as a potential therapy for wound healing.

**Supplementary Information:**

The online version contains supplementary material available at 10.1186/s12906-022-03737-4.

## Background

Propolis is a resin-like material made by bees that is considered one of the oldest herbal extracts used in medical remedies. Its unique properties are based on the rich flavonoids, phenols, and essential oils it contains, which grant it potent antimicrobial [1], anti-inflammatory [2], and antioxidant [3, 4] properties, making it effective during tissue healing. Furthermore, the propolis extract (propolis-EXTR) has enhances stem cell biological activities such as proliferation, migration, and chondrogenic and adipogenic differentiation [5].

Previously, propolis paste was used to accelerate wound healing in an experimental dog study in which the author reported a significant result compared to the control group [6]. However, in their study, propolis was used at a very high concentration, where treatment was administered twice daily until the end of the experiment (28 days). Similarly, a randomized controlled clinical trial has recently shown that propolis effectively accelerated wound healing after uncomplicated sacrococcygeal pilonidal cyst treated with marsupialization. However, a significantly reduced wound area was not reported before 28 days [7].

Studies have reported that one of the promising ways to promote the efficacy of herbal extracts is to incorporate them into nanostructures [8–10]. Therefore, nanostructured lipid carriers (NLCs) are novel drug delivery systems based on blends of lipids with nanoscale, high surface area to volume ratio that facilitates the cellular uptake of products overcomes the skin barrier restrictions, targets the epidermis, and reduces systemic absorption side effects [11]. For example, a previous study encapsulated propolis in solid lipid nanoparticles (SLNs) (particle size = roughly 111.3 nm). As a result, it reduces skin edema by enhancing skin permeation with no cytotoxic effects [12]. More recently, propolis-EXTR loaded on polymeric nanoparticles revealed a significant effect in reducing wrinkles and increasing collagen production by 25% [13].

Therefore, this study evaluated the efficacy of natural-based nanoparticles loaded with propolis in healing full-thickness skin wounds in rabbits. We also examined whether propolis-NLCs had a broad-spectrum antimicrobial effect than the previously established narrow-spectrum antibacterial efficacy of propolis-EXTR.

## Materials and methods

### Chemicals and reagents

The natural propolis powder was originally from Southeast Asia which purchased from a nature shop (Imtenan health shop; Cairo, Egypt). However, ascorbic acid, capric acid, glycerol mono-stearate (GMS), lecithin, sodium carbonate, tween 80, tannic acid, Carbopol-942, triethanolamine, and propylene glycol were purchased from EL-Gomhouria. This company trades in chemicals and medical appliances (Alexandria, Egypt). Furthermore, while 2,2-Diphenyl-1-picrylhydrazyl (DPPH), Folin-ciocalteu (Folin-C) phenol reagent, and dimethyl sulfoxide were purchased from Sigma-Aldrich Co. (ST. Louis, MO, USA), ethanol and methanol were also purchased from EL-Gomhouria. Additionally, anaesthetic drugs used in surgery such as Xylaject was obtained from Adwia Co. (Egypt), whereas Ketamine Hydrochloride was obtained from Rotexmedica (Germany).

### Preparation of propolis-EXTR

**Fig. 1:**
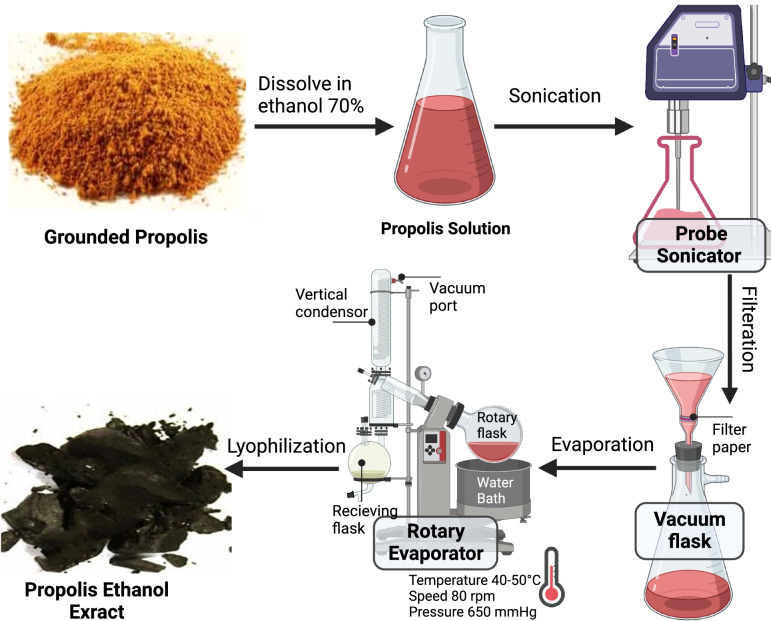
Hydro-alcoholic extraction of raw grounded propolis

The propolis extract was prepared according to a previously reported protocol [14], where 120 g of propolis powder in 1380 mL ethanol 70% was sonicated for three hours at room temperature (Fig. 1). Subsequently, the mixture was filtered and evaporated under reduced pressure in a rotary evaporator with a water bath temperature of 40 °C –50 $^\circ \mathrm{C}$, pressure of 650 mmHg, and a speed of 80 rpm. The final product was lyophilized using a Christ Alpha 1–2 LD plus Freeze Dryer (Germany) for 48 h [15]. Finally, the percentage of extraction yield was calculated using the following equation:

$$\mathrm{Yield}\;(\%)=\frac{\mathrm{Weight}\;\mathrm{of}\;\mathrm{propolis}\;\mathrm{extract}\;\left(\mathrm g\right)}{\mathrm{Weight}\;\mathrm{of}\;\mathrm{raw}\;\mathrm{propolis}\;\mathrm{powder}\;\left(\mathrm g\right)}\times100.$$

### Preparation of propolis-NLCs

**Fig. 2:**
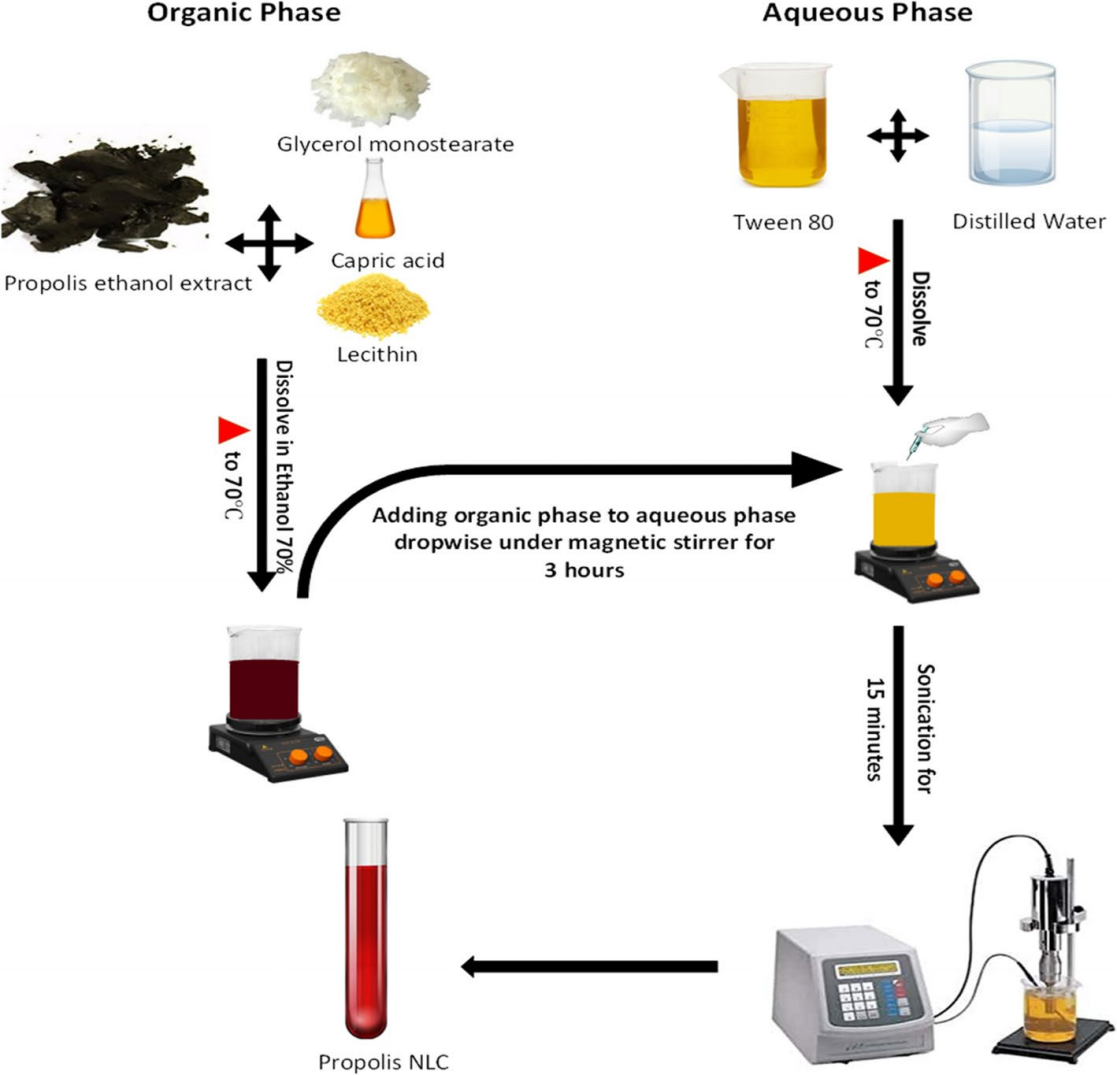
Preparation of Propolis Nanostructured Lipid Carriers (NLCs)

Propolis-NLCs were prepared using the emulsion-evaporation-solidification method previously reported by Chen et al. [16] with some modifications (Fig. 2). First, three propolis concentrations were dissolved in lipids (GMS & capric acid) and lecithin in 70% ethanol, then heated at 70 $^\circ \mathrm{C}$ for the formation of the organic phase (Table 1). However, for the aqueous phase formation, tween-80 was dissolved in distilled water and heated to 70 $^\circ \mathrm{C}$. Subsequently, the organic phase was drop-wisely to the aqueous phase at 70 $^\circ \mathrm{C}$ under a magnetic stirrer for three hours. Then, the reaction suspension was sonicated for 15 min using a high-energy ultrasonic device. This device comprised an ultrasonic probe (an HD 2070 ultrasonic Homogenizers with a HF generator (GM 2070)), ultrasonic converter (UW2070), a booster horn (SH 213 G), and a probe microtip (MS 73, Ø 3 mm), and the reaction condition was at 7 kHz and 9 cycles/sec, controlled by the device’s software, finally producing the propolis-NLCs.

**Table 1:** Propolis nanostructured lipid carrier (Propolis-NLC) formulations

**Components**	**Amount**
Propolis	25, 50, or 75 mg
Glycerol mono-stearate (GMS)	0.7 g
Capric acid	0.3 mL
Lecithin	0.5 mL
Ethanol	1.5 mL
Tween 80	0.2 mL
Distilled water	6.75 mL
Total sample	10 mL

Organic phase: Glycerol mono-stearate (solid lipid), capric acid (liquid lipid), lecithin and ethanol. Aqueous phase: Tween 80 and distilled water

### Characterization of propolis-NLCs

#### Scanning electron microscopy (SEM)

Propolis-NLCs particle sizes were visualized using a JEOL JSM-IT200 InTouchScope™ SEM (Inc., Japan). First, samples were suspended in distilled water by ultrasonication for 15 min. Then, they were loaded on the metal stubs with a double-sided tape and coated with gold before imaging.

#### Transmission electron microscopy (TEM)

Propolis-NLCs were suspended in ethyl alcohol by sonicating for 15 min. Then, a drop of propolis-NLCs was placed in a copper grid coated with thin carbon films. Subsequently, the grid was transferred to a specimen holder for microscopic evaluations using a JEOL JEM-1400 plus TEM (Inc., Japan).

#### Zeta potential (ZP) and polydispersity index (PDI)

Propolis-NLCs samples were inspected through photon correlation spectroscopy (Zetasizer Nano ZS, Malvern Instruments, USA). Then, measurements were conducted after diluting with distilled water and sonicating. After that, the PDI values were obtained using polystyrene cells at a 25 $^\circ \mathrm{C}$ and refractive index (RI) to measure lipid nanoparticles’ dispersion = 1.330 (abs = 0.01). ZP was subsequently performed using zeta-dip cells for 12 runs, demonstrating the charge presented on the surface of the nanoparticles and reflecting their physical stability [17].

#### Entrapment efficiency (EE) and drug loading (DL)

EE is the percentage of drugs integrated into lipid particles relative to the total drug added. However, DL is the percentage of drugs incorporated into lipid nanoparticles relative to the total weight of the lipoidal phase. Propolis-NLCs were centrifuged at 5000 rpm for 20 min and at a wavelength of 323 nm. Then, all wavelengths were detected through the spectrum after which standard curves were produced after measurements [18, 19]

The following equations were used for calculating EE (%) and DL (%):

$$\mathrm{EE}\;(\%)=\frac{\mathrm{Amount}\;\mathrm{of}\;\mathrm{drug}\;\mathrm{added}\;-\;\mathrm{Amount}\;\mathrm{of}\;\mathrm{the}\;\mathrm{drug}\;\mathrm{in}\;\mathrm{the}\;\mathrm{supernatant}}{\mathrm{Amount}\;\mathrm{of}\;\mathrm{drug}\;\mathrm{added}}\times\;100$$

$$\mathrm{DL}\;(\%)=\frac{\mathrm{Amount}\;\mathrm{of}\;\mathrm{drug}\;\mathrm{added}\;-\;\mathrm{Amount}\;\mathrm{of}\;\mathrm{the}\;\mathrm{drug}\;\mathrm{in}\;\mathrm{the}\;\mathrm{supernatant}}{\mathrm{Amount}\;\mathrm{of}\;\mathrm{drug}\;\mathrm{added}\;+\;\mathrm{Amount}\;\mathrm{of}\;\mathrm{excipients}\;\mathrm{added}}\times100$$

### Determination of the total phenolic content of propolis-EXTR and propolis-NLCs

Phenolic content estimation was determined following the Folin-C method as previously described [9, 20, 21]. A standard curve was finally produced using tannic acid solutions ranging from 10 to 80 mg/L (*r*^2^ = 0.9902). First, the stock solution of each preparation was prepared by dissolving 0.01 g into 5 mL of distilled water. Secondly, 250 µL of each stock was dissolved in ethanol 95% (250 µL), 1250 µL distilled water, and 125 μL Folin-Ciocalteu reagent (1 N). The mixture was left at room temperature for 5 min before adding 250 µL sodium carbonate solution (5%, w/v). Finally, the blank was prepared similarly but without tannic acid. After one hour of incubation in darkness, the absorbance was measured using a UNICO-1200 spectrophotometer at a wavelength of 760 nm. The total phenolic content was expressed in milligrams per gram of tannic acid equivalent.

### Determination of total flavonoid content of propolis-EXTR and -NLCs

According to previous studies [22, 23], the flavonoid content was determined by building a calibration curve using rutin reference solutions ranging from 50 to 300 mg/L (*r*^2^ = 0.9955). While rutin-prepared solutions (0.5 mL) were first added to 0.5 mL aluminum chloride (20 mg/mL) solution, the blank was prepared by diluting aluminum chloride with ethanol. Next, the respective solutions were incubated for one hour at room temperature. Absorbance measurements were finally taken in triplicate (λ = 420 nm). For propolis-EXTR and propolis-NLCs, samples were prepared following the same routine used for the reference solutions. Then, total flavonoid content was calculated as g/g rutin equivalents.

### Determination of the antioxidant activity of propolis-EXTR and -NLCs

The DPPH radical scavenging activity was estimated depending on the ability of antioxidant compounds to lose hydrogen to the DPPH radical [24, 25]. Therefore, different concentrations of each sample were prepared with methanol. Then, 1 mL of 0.2 mM DPPH was added to 1 mL of the prepared concentrations, then stirred. After ten minutes of incubation in darkness, the absorbance was measured (λ = 517 nm). All determinations were performed in triplicate. Subsequently, serial dilutions of ascorbic acid were also prepared for the calibration curve (*r*^2^ = 0.966) after which the percentage of the DPPH scavenging activity was calculated as follows:

$$Inhibition\;(\%)=\frac{\mathrm{Absorbance}\;\mathrm{of}\;\mathrm{control}\;-\;\mathrm{Absorbance}\;\mathrm{of}\;\mathrm{sample}}{Absorbance\;of\;control}\times\;100$$

### Encapsulation of propolis-EXTR and-NLCs into carbopol gels

High antioxidant activity and ZP value (75 mg/10 mL) parameters were selected for the skin excision. According to Z Naz and FJ Ahmad [26], 4 mL of propolis-EXTR and propolis-NLCs solution were added to 0.5 g Carbopol-942 under a magnetic stirrer. Then, to assess the neutralization and plasticity of the gel, 0.5 mL triethanolamine and a few drops of propylene glycol were integrated into the dispersion to obtain a final volume of 50 g of gel (0.6 mg/mL). For *in vivo* studies, a blank gel was prepared using the same procedure without adding the understudied compounds.

### Evaluation of the antimicrobial properties of the carbopol encapsulated propolis-EXTR and propolis-NLCs

The disk diffusion method for testing the antimicrobial activity of formulated carbopol encapsulated propolis-EXTR and -NLCs using the clinical and laboratory standards institute (CLSI) method was performed [27, 28]. First, five bacterial culture strains, including *Bacillus subtilis* American Type Culture Collection (ATCC) 6633, *Escherichia coli* ATCC 25922, *Salmonella spp.*, *Staphylococcus aureus* ATCC 25923, *Staphylococcus epidermis,* and one fungal strain culture, namely, *Candida albicans* EMCC 105, were used according to their availability and relationship to the animal wound infection. All used strains in this study were kindly provided from the American Type Culture Collection (ATCC), USA. Next, bacterial strains were activated overnight in nutrient broth at 37 $^\circ \mathrm{C}$ and yeast mold broth at 25 $^\circ \mathrm{C}$, respectively, until they reached 1 × 10^5^ colony-forming units (CFU/mL). Subsequently, 1 mL of the inoculum was transferred aseptically to pre-sterilized Petri dishes, after which the liquefied culture medium (45 °C–50 °C) was poured on the inoculum. Then, sterile 5 mm disc papers were saturated separately from the pre-formed gels (500 mg/mL) and placed on the media surface. Also, the plates were incubated overnight at 37 $^\circ{\rm C}$ for bacteria and 25 $^\circ \mathrm{C}$ for the tested fungus. After that, the ability of the tested gels to inhibit microbial growth was finally determined by measuring the diameter of the inhibition zone. The experiment was performed in triplicate, and the average values were tabulated.

The minimal inhibitory concentration (MIC) is the lowest concentration of an antimicrobial to inhibit the visible growth of a microorganism. As described by Humphries et al. [27], the MIC was detected using the disk diffusion method for all diluted solutions. Therefore, a high concentration of crude extract (500 µg/mL) was prepared as stock solution and serially diluted (2-fold), ranging from 3.9 to 500 µg/mL.

### *In vivo* full-thickness skin animal injury and treatment protocol

All animal care, handling, and study were conducted in the laboratory animal research department at the Faculty of Agriculture, and Faculty of Veterinary Medicine, Alexandria University, according to the approved protocols by the institutional animal care and use committee (AU-IACUC). Eighteen male rabbits (New Zealand, weighing 1.9–2.0 kg) were randomly fractionated into three groups. Then, skin excision surgeries were conducted under the effect of general anaesthesia in which the animals were sedated by xylazine (5 mg/kg) followed by ketamine (35 mg/kg). Each rabbit had two full-thickness skin excisions (3 × 3 cm) on its dorsum, receiving different treatments on each side for seven days after the surgery, as shown in our previous protocol [29]. Accordingly, after surgery, the animals were injected with 0.2 mL of the anti-inflammatory drug (meloxicam) on the second day. Next, the wounds were randomly allocated into three groups: the carbopol alone (*n* = 9), propolis-EXTR @Carbopol gel (*n* = 9), and propolis-NLCs @Carbopol gel (*n* = 9) groups. After applying different treatments topically, wounds were covered with a hypoallergenic adhesive nonwoven sterile wound dressing with an absorbent wound pad. The wounds were finally followed until complete healing (three weeks).

#### Wound healing assessment

The wounds were photographed from the first day of wound induction then followed up on days 7, 14, and 21 using a digital camera (Nikon D5100, Japan). Subsequently, measurements were taken using the ImageJ software, after which the wound surface areas were calculated as the percentage of wound healing using the following equation:

$$Wound\;healing\;(\%)=\frac{\mathrm{Initial}\;\mathrm{day}\;\mathrm{wound}\;\mathrm{size}\;-\;\mathrm{Specific}\;\mathrm{day}\;\mathrm{wound}\;\mathrm{size}}{\mathrm{Initial}\;\mathrm{day}\;\mathrm{wound}\;\mathrm{size}}\times\;100$$

#### Histopathological evaluation

Histopathological specimens, including the healed skin and surrounding healthy skin in all groups, were collected on day 14 of drug application. Then, the samples were washed with phosphate buffer saline and fixed in 10% neutral buffered formaldehyde for 48 h. Subsequently, the fixed specimens were processed using the conventional paraffin embedding technique and embedded in paraffin wax. Next, the embedded samples were cut into 4 µm thick sections and stained later with Hematoxylin and Eosin (H&E) dyes according to the method described by JD Bancroft and C Layton [30]. Also, some sections were stained with Masson’s trichrome for total collagen detection. Stained sections were finally visualized using a microscope (Leica DM500) equipped with a digital camera (Leica EC3, Leica, Germany).

## Statistical analysis

*In vitro* and *in vivo* experimental data were collected from three independent experiments after which all statistical analyses were performed using GraphPad software version 8.0 (La Jolla, CA, USA). The results were reported as mean ± standard deviation (SD). Subsequently, 2-way ANOVA followed by Šídák's multiple comparisons was conducted for the comparisons between different groups. Differences were considered significant at * *p* < 0.05, ** *p* < 0.01, *** *p* < 0.001, and **** *p* < 0.0001.

## Results and discussion

### Extraction of raw ground propolis

Propolis acts as an anti-inflammatory, antioxidant, and antimicrobial agent during wound healing because of its organic components, such as flavonoids and polyphenols. Since it has been reported that those substances are highly soluble in aqueous 70% ethanol over water and oils, these active components were extracted with 70% ethanol (Fig. 1). The alcoholic extraction produced a brown mass of propolis with a yield of 22.10%, Then the propolis-EXTR was stored in the dark at less than 25 °C [31].

Propolis is a natural resinous honeybee product containing 50–55% resin, 30% beeswax, 10% naphtha, and some pollen [32]. Chemically, it is very complex, and the components vary significantly according to its geographical and botanical origins [33, 34]. Propolis significantly impacts the appreciation of its organoleptic character through its volatile fraction, which is one of the essential quality attributes [35]. Different classes of volatile compounds, such as aldehydes, ketones, alcohols, esters, terpenes, and acids, are found in propolis [36]. The detailed phytochemical components of propolis and its nanoparticles would be investigated in the future.

### Nanostructured lipid carrier preparation

The emulsion-evaporation-solidification technique was used to form propolis-NLCs (Fig. 2). This method adopts organic and aqueous phase heating at 70 °C to melt lipids in the mixture and reduce the solvent/water content. Subsequently, while GMS was the solid lipid due to its ability to solidify and emulsify oils, waxes, and solvents, capric acid considered a medium-chain triglyceride with rapid body absorption, was the liquid lipid. This blending of lipids enhanced propolis loading capacity and minimized its expulsion during storage. Alternatively, lecithin and tween 80 acted as a homogenizer, stabilizers, and surfactant agent, yielding semi-solid nanoemulsions subjected to sonication and gelatinization for topical application.

### Characterization of propolis-NLCs

**Fig. 3:**
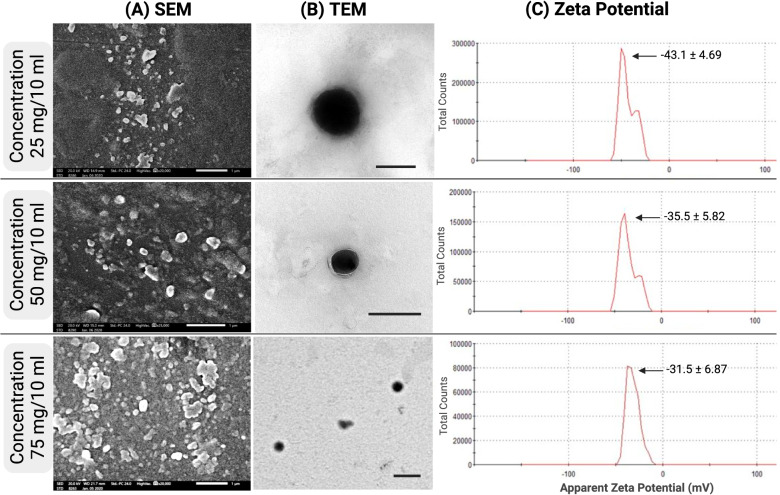
Characterization of Propolis-NLCs at 3 different concentrations 25, 50 and 75 mg. **A** Scanning electron microscopy (SEM) of Propolis-NLCs, Scale bar = 1 µm. **B** Transmission electron microscopy (TEM) of Propolis-NLCs, scale bar = 200 µm. **C** Zeta potential (ZP) of Propolis-NLCs. The zeta potential was tested at a temperature of 25 °C; zeta runs = 12 using clear disposable zeta cells

SEM images showed a uniform elliptical shape for propolis-NLCs (Fig. 3A). Measuring the propolis-NLCs shape and size of different concentrations revealed that the mean particle sizes ranged from 41.57 to 44.28 nm (Table 2, Fig. 3A). Furthermore, TEM images (Fig. 3B) illustrated the morphology of the prepared nanoparticles, which werecompatible with the scanning microscopy results. As perviously reported by Larese Filon et al. [37],although this particle size range cannot penetrate intact skin, it can penetratethe damaged skin. Besides, there are no reported side effects from propolis nanoparticles and are no considered hazardous materials compared to metal nanoparticles have been recorded. Based on these facts, the small particle size used in this study is advantageous for delivering the active principles to the deep tissues. Moreover, studies have previously reported that this small particle size can be attributed to the use of a 7% emulsifier and a high surfactant concentration of 7% [38, 39].

**Table 2:** Characterizations of Propolis-NLCs formulations

**Formulation**	**Propolis weight (mg)**	**Particle size (nm) ± SE**	**ZP** **(mV) ± SD**	**PDI**	**EE (%) ± SD**	**DL (%) ± SD**
1	25	41.57 ± 1.96	-43.1 ± 4.69	0.348	87.21 ± 0.79	1.43 ± 0.01
2	50	44.28 ± 2.32	-35.5 ± 5.82	0.393	85.03 ± 0.18	2.75 ± 0.02
3	75	41.64 ± 0.85	-31.5 ± 6.87	0.290	83.29 ± 0.47	3.97 ± 0.02

Particle size as detected by scanning microscope on scale 500 nm

*ZP* Zeta potential, *PDI* Polydispersity index, *EE* Entrapment efficiency, *DL* Drug loading of Propolis-NLCs formulations

Photon correlation spectroscopic results for the ZP and PDI for propolis-NLCs ranged between − 31.5 ± 6.87 to − 43.1 ± 4.69 mV and 0.290 to 0.348 respectively (Table 2, Fig. 3C). This study also illustrated that while the long-term stable dispersions had high ZP values as 75 mg/10 mL concentration formulation, for formulations of low PDI, the values were greatly homogeneous in the distribution (Table 2).

### EE and DL

Subsequently, to study EE and DL, spectrum density and standard curves were used for the main compounds to adjust their optimal wavelengths and calculate their concentrations. Then, standard curves were drawn, using an optimum absorbent wavelength of 323 nm for propolis. The regression equations were spotted as follows: Y = 0.0037x, *R*^2^ = 0.9828. Results showed that while the EE of propolis-NLCs (75 mg) was 83.29% ± 0.47%, the DL was 3.97% ± 0.023. However, when it increased to 85.03% ± 0.18%, DL was 2.75% ± 0.021% in propolis-NLCs (50 mg) and 87.21% ± 0.79% with 1.43% ± 0.013% in propolis-NLCs (25 mg) (Table 2). Previously, Chen et al. [33] revealed that increasing the drug to lipid ratio reduces the DL capacity and diminishes the drug EE. Accordingly, this study showed that propolis-NLCs with high drug concentrations possessed a high percentage of DLs that lowered the drug EE.

### Total phenolics, total flavonoids, and antioxidant activities

**Fig. 4:**
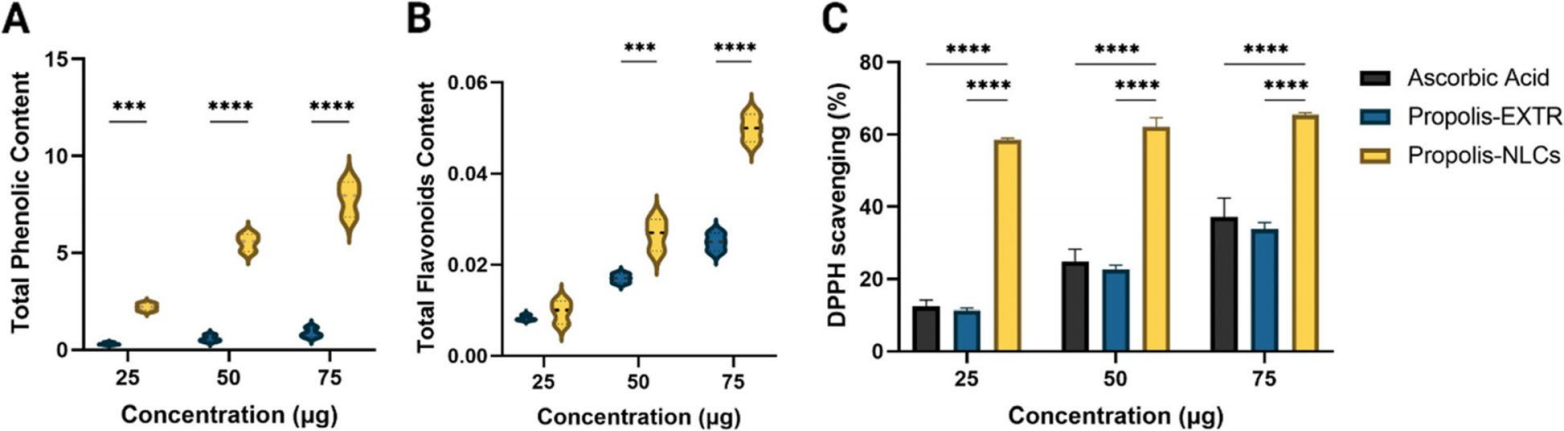
Phytochemical content of Propolis-EXTR and Propolis-NLCs. **A** Total phenolic content and (**B**) Total flavonoid content at three different concentrations 25, 50, and 75 mg. The antioxidant activity of Propolis-EXTR and Propolis-NLCs compared to ascorbic acid using quantitative analysis by DPPH scavenging activity test (**C**). Data represented as mean ± SD (***p < 0.001, ****p < 0.0001)

The natural products’ antioxidant activity and their phenolic and flavonoid contents clarify the direct relationship between these contents and their reducing activity. Our results showed that the propolis-NLCs 75 mg had 7.83 ± 0.9 phenolic content compared to 0.88 ± 0.25 mg tannic acid equivalent (mg/g) in propolis-EXTR (~ 9-Fold, *p* < 0.0001, Fig. 4A). Furthermore, propolis-NLCs amplified the content of flavonoids two-fold higher than propolis-EXTR (*p* < 0.0001, Fig. 4B). Notably, investigations also revealed that increasing the concentration in propolis-NLCs from 25 to 75 mg revealed a significant increase (*p* < 0.0001) in the phenolic and flavonoid contents, estimated by 3.5-fold and 4.7-fold, respectively.

The high reactive oxygen species (ROS) and oxidative stress level due to cell damage at the injury site could impair healing processes. Hence, a wound dressing with antioxidants is essential to fasten the wound healing rate and quality [40]. Therefore, the DPPH scavenging technique was used to investigate the antioxidant activity of propolis-EXTR and -NLCs. The results indicated that the propolis-NLCs possessed a potent antioxidant activity compared with ascorbic acid (*p* < 0.0001) and propolis-EXTR (Fig. 4C). The propolis-NLCs exhibited DPPH inhibition of 58.50, 62.07, and 65.47% for the samples containing 25, 50, and 75 mg propolis, respectively. It has also been reported that the antioxidant activity of the propolis-NLCs was attributed to their high content of phenolic and non-phenolic compound contents that could preventively attack free radicals during the oxidation reaction [41, 42]. In addition, data revealed that the transformation of propolis-EXTR to nanoparticles improved their scavenging activity a highly significant difference compared to their native formulations.

### Antimicrobial and antifungal activities

**Fig. 5:**
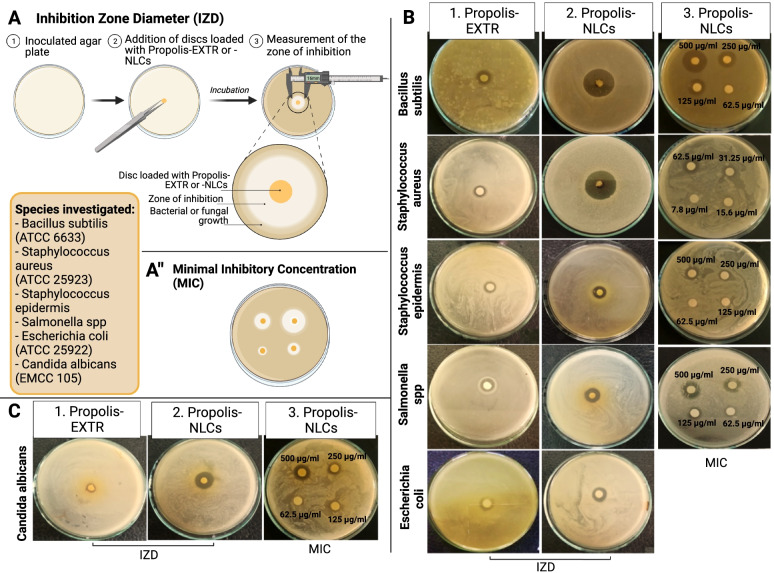
Assessment of the antibacterial and antifungal activity of Propolis-EXTR and Propolis-NLCs. Schematic diagram of the experiments (**A**) inhibition zone diameter and (**A "**) minimal inhibitory concentration. **B** Antibacterial activity of Propolis-EXTR and Propolis-NLCs against different bacterial species as Bacillus subtilis ATCC 6633, Staphylococcus aureus ATCC 25923, Staphylococcus epidermis, Salmonella spp, and Escherichia coli ATCC 25922 using IZD (1 & 2) and MIC (3). **C** Antifungal activity of Propolis-EXTR and Propolis-NLCs against Candida albicans EMCC 105

The propolis’ antimicrobial activity was proposed through two mechanisms. First, it directly affects the bacterial membrane and inhibits its motility. Then, it stimulates the immune system [43, 44]. The antimicrobial activity and MIC of propolis-EXTR and -NLCs @carbopol gels were evaluated against five bacterial strains and one fungal strain using the agar disk diffusion method. First, the antimicrobial activity was evaluated by measuring the inhibition zone diameter (IZD, mm, Fig. 5A). Simultaneously, the MIC was assessed using similar technique but by measuring the lowest amount of gel as a mg/ml solution that can inhibit microbial growth after 24 h of incubation (Fig. 5A).

The results showed that propolis-NLCs @carbopol gel had a potent antimicrobial activity against similarly tested bacterial strains. For example, Table 3 and Fig. 5B clarified that propolis-NLCs exhibited 26 mm IZD *B. subtilis ATCC 6633* with 125 µg/mL MIC, 27 mm IZD for *S. aureus* ATCC 25,923 with 31.25 µg/mL MIC, and 18 mm IZD against *Salmonella spp.* with 125 µg/mL. Similarly, the IZD was 14 mm for *E. coli* ATCC 25,922 and *S. epidermis* with MIC 500 µg/mL and 250 µg/mL successively.

**Table 3:** Antimicrobial activity evaluation of the formulated gels

**MICROBIAL STRAINS**	**PROPOLIS-EXTR @CARBOPOL**		**PROPOLIS-NLCS @CARBOPOL**	
	**MIC (µg/mL)**	**IZD (mm) ± SD**	**MIC (µg/mL)**	**IZD (mm) ± SD**
*B. subtilis ATCC 6633*	250	12 ± 0.87	125	26 ± 1.04
*S. aureus* ATCC 25923	500	10 ± 0.87	31.25	27 ± 1.25
*S. epidermis*	500	12 ± 0.56	250	14 ± 0.89
*Salmonella SPP*	500	10 ± 0.46	125	18 ± 1.47
*E. coli* ATCC 25922	500	13 ± 0.87	500	14 ± 0.96
*C. albicans* EMCC 105	500	9 ± 0.46	125	16 ± 0.92

*MIC* Minimal inhibitory concentration, *IZD* Inhibition zone diameter

Contrastively, we observed that the gel containing the propolis-EXTR manifested a lower antibacterial activity against the tested strains. For example, it exhibited 13 mm IZD against *E. coli* ATCC 25,922 with 500 µg/mL MIC, 12 mm for *B. subtilis ATCC 6633,* and *S. epidermis* with 250 µg/mL and 500 µg/mL MIC in turn. It also showed 10 mm IZD with 500 µg/mL MIC against both *Salmonella spp.* and *S. aureus* ATCC 25,923 (Fig. 5B).

Similar to previous reports, these results also demonstrate that propolis-EXTR had potent antibacterial effects against Gram-positive bacteria such as *B. subtilis* and *S. aureus* but not the Gram-negative bacteria (*Salmonella spp*) [43]. Specifically, while propolis-NLCs increased in antibacterial effect against Gram-positive bacteria more than twofold compared with EXTR, they interestingly, has a strong effect on the Gram-negative *Salmonella spp* (1.8-fold, *p* < 0.0001, Table 3).

The antifungal activity of the formed gels is demonstrated in Fig. 5C and Table 3. Investigations revealed that the propolis-NLCs @carbopol gel showed the highest antifungal activity against *C. albicans* EMCC 105 with 16 mm IZD and 125 µg/mL MIC. However, the antifungal activity obtained from the propolis-EXTR @Carbopol gel was significantly lower, as it revealed 9 mm IZD 500 µg/mL MIC (*p* < 0.0001, Table 3).

### Efficiency to improve the healing rate and quality of full-thickness skin wounds

**Fig. 6:**
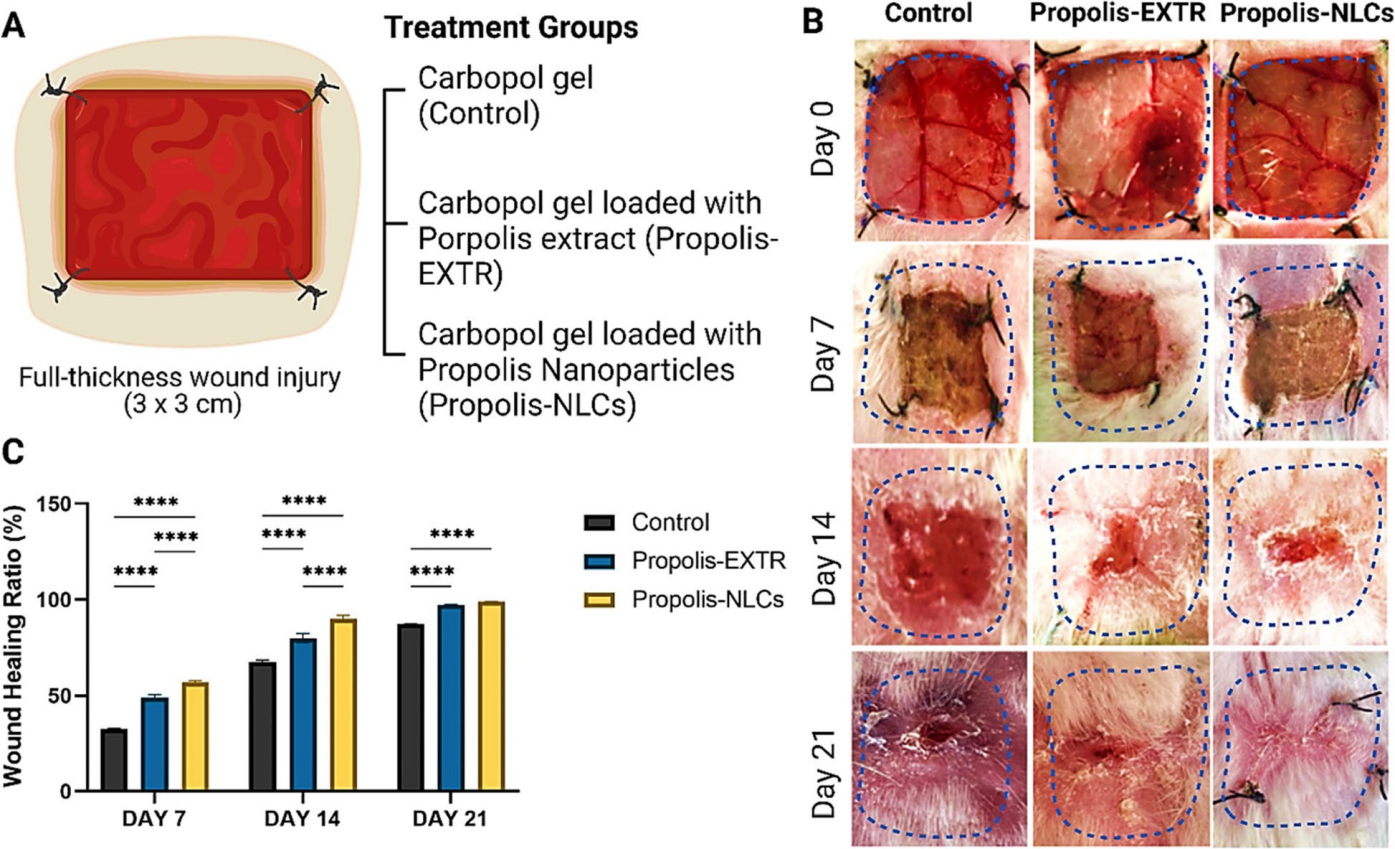
Assessment of wound healing efficiency of Propolis-EXTR and Propolis-NLCs in comparison to control group treated with Carbopol alone. **A** Schematic diagram of the *in vivo* full-thickness skin injury experiment design in rabbits and the treatment groups. **B** Representative images show wound dimensions. **C** Qualitative analysis of the wound healing ratio in control, Propolis-EXTR, and Propolis-NLCs groups after induction of injury (0 day) and then after 7, 14, and 21 days. Data represented as mean ± SD (****p < 0.0001)

While carbopol as a loading gel alone was considered the control, propolis-EXTR and NLCs -loaded carbopol were applied to treat full-thickness skin defects in rabbits (Fig. 6A). The nanotreated wounds showed rapid wound healing in all groups with healthier skin formation and without oozing or necrosis (Fig. 6B). However, only a slight difference in wound healing was observed between the propolis-EXTR @carbopol gel-treated wounds and blanked carbopol gel. Comparatively, propolis-NLCs @carbopol gel-treated wounds showed a significantly higher wound healing rate (56.97%) than propolis-EXTR @carbopol gel (49.10%) on day 7 (*p* < 0.0001, Fig. 6C). Results also showed that although the propolis-NLCs @carbopol gel-treated wounds reached a 90.03% healing rate by day 14, propolis-EXTR @Carbopol gel was 79.92% (*p* < 0.0001, Fig. 6C).

The high antioxidant efficiency of propolis-NLCs can explain their accelerated healing rate, as shown in this study. Previously, Fitzmaurice et al. [40] reported that the high level of ROS resulting from the damaged tissue could delay healing rates. *In vitro* experiments, in a previous study also showed that propolis-EXTR could enhance stem cell proliferation and migration [5]. Similarly, another group demonstrated the positive effect of propolis on keratinocyte migration and wound closure using the scratch assay [45]. Hence, propolis-NLC is a promising therapy.

**Fig. 7:**
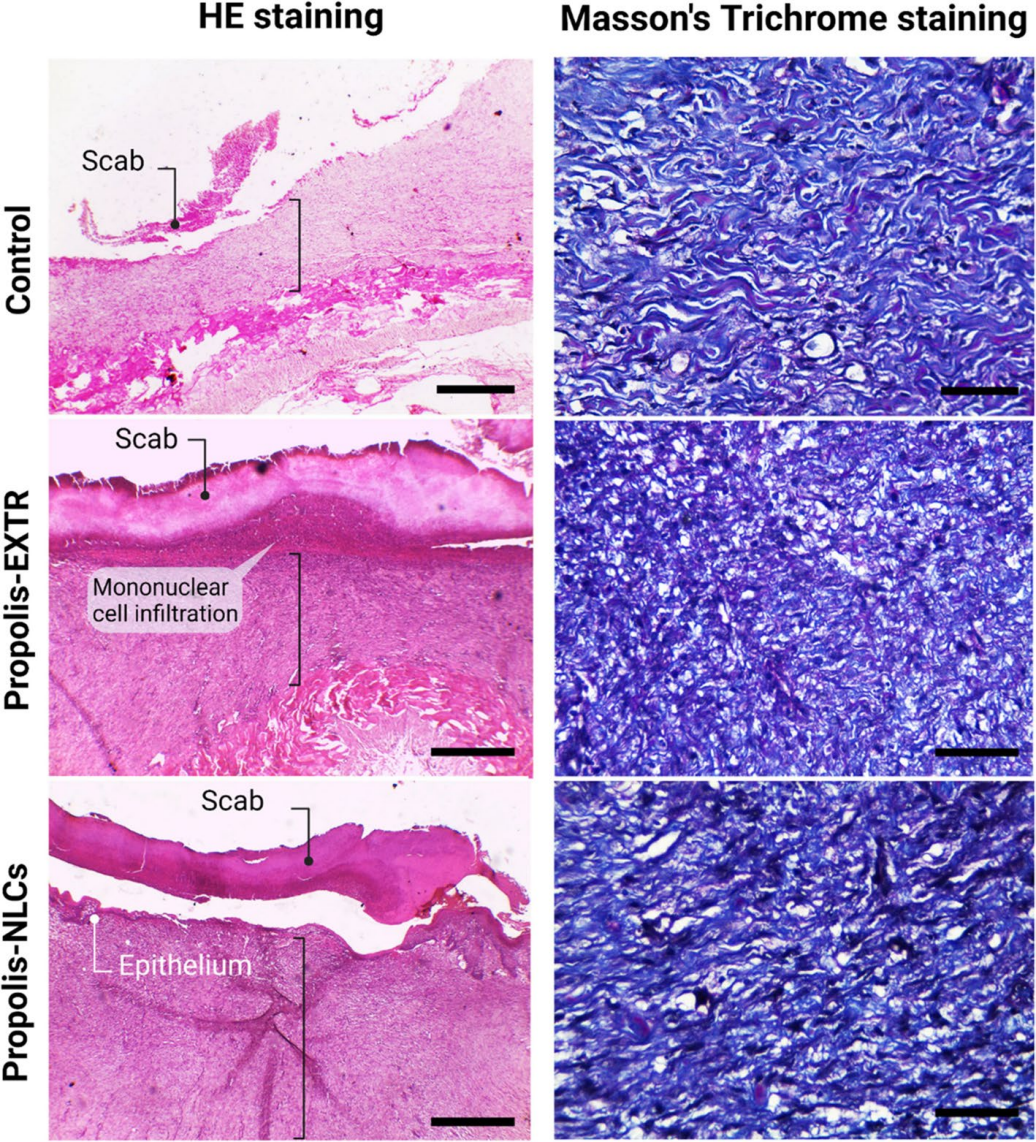
Histopathological examination of skin treated with propolis-EXTR and propolis-NLCs after 14 days. The left panel shows representative H&E images for the control wounds treated with Carbopol gel (control), propolis-EXTR, and propolis-NLCs loaded on Carbopol gel. The images reveal scab formation (black round tip arrow) and epithelium formation (white round tip arrow) under the scab. Scale bar = 400 µm. The right panel shows representative images for Masson Trichrome staining for the control wounds treated with Carbopol gel (control), propolis-EXTR, and propolis-NLCs loaded on Carbopol gel. Scale bar = 50 µm

During the first week, histopathological evaluations of skin injury in the control carbopol gel group revealed coagulative necrosis (scab formation) with mild fibroblastic proliferation on day 7. However, propolis-EXTR @ and propolis-NLCs @carbopol gels displayed scabs formed by necrotic tissue remnants with mononuclear cell infiltration and immature granulation tissues (Fig. S1). In the second week, while histological findings of the carbopol gel group revealed thickened skin covered by a mature granulation tissue, in the propolis-EXTR and -NLC groups, healed skin showed complete epidermal regeneration under the scab and increased skin thickness, particularly in wounds treated with propolis-NLCs (Fig. 7). In the third week, both propolis-EXTR and propolis-NLCs showed a complete epidermis formation. However, the propolis-EXTR showed scanty haemorrhage in the dermis layer (Fig. S2). Subsequently, Masson trichrome staining was used for further histological investigations. Images of propolis-EXTR and propolis-NLCs revealed early mature granulation tissues with mixed vertical and horizontal orientations, including mild to moderately new blood vessels (Fig. 7).

## Conclusion

Nanotechnology can provide a platform for maximizing the benefits and increasing propolis-EXTR efficiency through the lipid nanocarrier technique. In this study, wound evaluation indicated that propolis-NLCs loaded on carbopol gel could successfully accelerate the healing of the wound compared with propolis-EXTR. Results also showed that while the negative control group was in the early stage of healing, propolis-NLCs could still provided fast wound closure, protection from microbial contamination, and minimal scar formation.

## Supplementary Information

**Additional file 1**: Histopathological examination of skin treated with propolis-EXTR and propolis-NLCs after 7 and 21 days.

## Data Availability

All data generated or analyzed during this study are included in this published article.

## Abbreviations

EXTR: Extract
NLCs: Nanostructured lipid carriers
SLNs: Solid lipid nanoparticles
GC/MS: Gas Chromatography/Mass Spectrophotometer
GMS: Glycerol mono-stearate
DPPH: 2,2-Diphenyl-1-picrylhydrazyl
SEM: Scanning Electron Microscopy
TEM: Transmission Electron Microscopy
ZP: Zeta potential; PDI, Polydispersity Index
EE: Entrapment efficiency; DL, drug loading
ATCC: American Type Culture Collection
CFU: Colony-forming units; MIC, minimal inhibitory concentration
PBS: Phosphate buffer saline
H&E: Hematoxylin and Eosin; SD, standard deviation
ROS: Reactive oxygen species
IZD: Inhibition zone diameter
