# Supplementary material for: Comparative evaluation of propolis nanostructured lipid carriers and its crude extract for antioxidants, antimicrobial activity, and skin regeneration potential
Source: BMC Complement Med Ther. 2022 Oct 3;22:256. doi: 10.1186/s12906-022-03737-4 (PMC9528112; doi:10.1186/s12906-022-03737-4)
Supplement: Supplementary file 1 — Additional file 1: Histopathological examination of skin treated with propolis-EXTR and propolis-NLCs after 7 and 21 days. [file 12906_2022_3737_MOESM1_ESM.docx]

**Supplementary Figures**


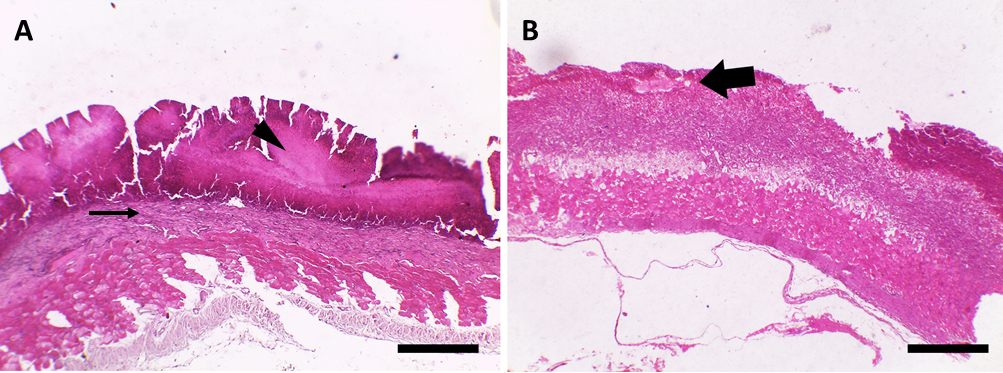


**Fig. S1.** Histopathological examination of skin treated with propolis-EXTR and propolis-NLCs after 7 days. Representative H&E images for propolis-EXTR (A) and propolis-NLCs loaded on Carbopol gel (B). The images reveal scab formation (arrowheads) and epithelium formation (thin arrow) under the scab; also, immature granulation tissue was noticed (thick arrow). Scale bar = 400 µm.


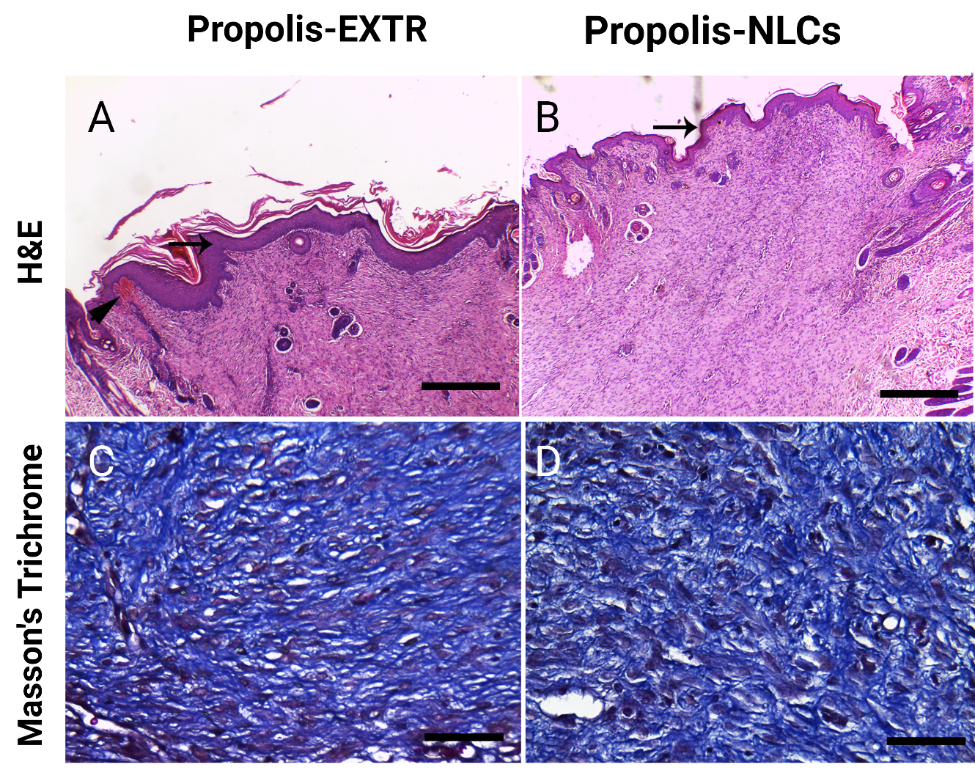


**Fig. S2.** Histopathological examination of skin treated with propolis-EXTR and propolis-NLCs after 21 days. (A & B) Representative H&E images for propolis-EXTR and propolis-NLCs loaded on Carbopol gel. The images show a complete epidermis (thin arrow) with scanty hemorrhage in the epidermis of the propolis group (arrowhead). Scale bar = 400 µm. (C & D) Representative Masson's Trichrome images for propolis-EXTR and propolis-NLCs loaded on Carbopol gel. Scale bar =50 µm.
